# Risk classification of patients referred to secondary care for low back pain

**DOI:** 10.1186/s12891-018-2082-y

**Published:** 2018-05-24

**Authors:** Monica Unsgaard-Tøndel, Ingunn Gunnes Kregnes, Tom I. L. Nilsen, Gunn Hege Marchand, Torunn Askim

**Affiliations:** 1 Department of Neuromedicine and Movement Science (INB), NTNU, Faculty of Medicine and Health Sciences, N-7491 Trondheim, Norway; 2 Department of Public Health and Nursing, Norwegian University of Science and Technology, Faculty of Medicine and Health Sciences, Trondheim, Norway; 3 Department of Physical Medicine and Rehabilitation, St. Olav’s Hospital, Trondheim University Hospital, Trondheim, Norway; 4 Clinic of Anaesthesia and Intensive Care, St Olavs Hospital, Trondheim University Hospital, Trondheim, Norway

**Keywords:** Primary care, Secondary care, Low back pain, Screening, Multidisciplinary, Work

## Abstract

**Background:**

Nonspecific low back pain is characterized by a wide range of possible triggering and conserving factors, and initial screening needs to scope widely with multilevel addressment of possible factors contributing to the pain experience. Screening tools for classification of patients have been developed to support clinicians. The primary aim of this study was to assess the criterion validity of STarT Back Screening Tool (STarT Back) against the more comprehensive Örebro Musculoskeletal Pain Questionnaire (ÖMPSQ), in a Norwegian sample of patients referred to secondary care for low back pain. Secondary aims were to assess risk classification of the patients, as indicated by both instruments, and to compare pain and work characteristics between patients in the different STarT Back risk categories.

**Methods:**

An observational, cross-sectional survey among patients with low back pain referred to outpatient secondary care assessment at Trondheim University Hospital, Norway. Cohen’s Kappa coefficient, Pearson’s r and a Bland-Altman plot were used to assess criterion validity of STarT Back against ÖMPSQ. Furthermore, linear regression was used to estimate mean differences with 95% CI in pain and work related variables between the risk groups defined by the STarT Back tool.

**Results:**

A total of 182 persons participated in the study. The Pearsons correlation coefficient for correspondence between scores on ÖMPSQ and STarT Back was 0.76. The Kappa value for classification agreement between the instruments was 0.35. Risk group classification according to STarT Back allocated 34.1% of the patients in the low risk group, 42.3% in the medium risk, and 23.6% in the high risk group. According to ÖMPSQ, 24.7% of the participants were allocated in the low risk group, 28.6% in the medium risk, and 46.7% in the high risk group. Patients classified with high risk according to Start Back showed a higher score on pain and work related characteristics as measured by ÖMPSQ.

**Conclusion:**

The correlation between score on the screening tools was good, while the classification agreement between the screening instruments was low. Screening for work factors may be important in patients referred to multidisciplinary management in secondary care.

## Background

Low back pain is the leading cause of years lived with disability globally [1, 2]. In addition to a negative impact on the individual’s health, it is associated with substantial financial costs, partly for management that is not supported by scientific evidence [3].

Management guidelines suggest that patients seeking care for nonspecific and uncomplicated low back pain should be offered treatment in primary care [4]. On the other hand, patients with possible indicators of serious pathology or with compound treatment needs due to complex psychosocial challenges should be referred to specialist health service for further investigation and treatment [5]. Specific causes for low back pain are uncommon, and for about 85% of patients, low back pain is defined as nonspecific since the pain does not seem to be connected to specific organic impairments [6]. Nonspecific low back pain is characterized by a wide range of possible triggering and conserving factors, including lifestyle, behavioral, biomechanical, and psychosocial influences [7, 8]. Therefore, initial screening needs to scope widely with multilevel addressment of possible factors contributing to the pain experience.

There are data suggesting that in general, previous management for patients with low back pain have failed to address its multifactorial nature and accordingly not contributed significantly to patients’ improvement in the long term [8]. Part of the reason for this may be that multifactorial and knowledge based assessment tools have not been available. However, screening tools for classification of patients have been developed to support clinicians when identifying the specific needs of individual patients. The STarT Back screening tool (STarT Back) is one such screening tool [9]. It contains nine items covering eight domains, which were selected based on established prognostic factors suggested to affect probability of recovery. STarT Back was originally validated in England [9], and has been tested and adapted in a range of countries including Belgium [10], Denmark [11, 12], Finland [13], China [14], Germany [15], Norway [16] and Sweden [17]. Most of these studies have been performed in primary care. Another screening tool is the more comprehensive Örebro Musculoskeletal Pain Screening Questionnaire (ÖMPSQ), which was developed in Sweden for early identification of patients at risk for developing a persistent back problem [18]. ÖMPSQ has been considered an appropriate reference standard since it is an established tool to support clinicians in identifying patients in need of more comprehensive treatment for low back pain [19]. Since the performance of screening tools is highly context dependent, testing the tool in varied clinical settings is necessary. Therefore we wished to explore whether risk estimation by STarT Back is comparable to ÖMPSQ in a Norwegian multidisciplinary secondary care setting.

The primary aim of this study was to assess the criterion validity of the STarT Back Screening Tool against the Örebro Musculoskeletal Pain Screening Questionnaire in a Norwegian sample of patients with low back pain referred to assessment at a university hospital. Secondary aims were to assess risk classification of the patients, as indicated by both instruments, and to compare pain and work characteristics between patients in the different STarT Back risk categories.

## Methods

### Design

An observational, cross-sectional survey was performed in an outpatient multidisciplinary clinic for musculoskeletal pain in Trondheim University Hospital, Norway.

### Participants

The study sample was patients referred for secondary line management because of low back pain. The inclusion criteria were as follows: Referred from their physician, manual therapist or chiropractor in the primary health care system with low back pain for more than 6 weeks. Exclusion criteria were age under 18, insufficient language capabilities, malignant disease, and unresolved social security or insurance problems.

### Variables

#### Background variables

Background variables included age, gender, marital status, country of birth, educational level, and work status. Participants that were not out of work, sicklisted or on work assessment allowance were classified as employed. We defined people with any percentage of sickleave as sicklisted. Information on other diseases category was also collected, including headache, pulmonary disease, coronary disease, hypertension, diabetes and an open category for diseases specified by the participants.

#### Measures

*STarT Back screening tool* contains nine questions, and was developed based on prognostic factors for longstanding disability due to low back pain [9]. The questions cover the following eight constructs: bothersomeness, referred leg pain, comorbid pain, disability, catastrophizing, fear, anxiety, and depression. Based on dichotomizing responses patients were given an overall tool score and a psychosocial subscale score. Patients were allocated to the high risk group if the psychosocial subscale score was ≥4, to the low risk group if the overall score was ˂ 4, and to the medium risk group if the overall score was ≥4. Participants with missing items on STarT Back screening tool were excluded from the analysis if risk classification could not be established as described in the original study [9].

*Örebro Musculoskeletal Pain Screening Questionnaire* (ÖMPSQ) was developed to assist health care providers in assessing risk of developing a persistent back problem. Originally it was aiming at predicting risk for work absenteeism due to sickness [20]. The scoring system ranges from zero to 210, with higher scores indicating a higher risk of poor outcome. It has shown good psychometric properties [18, 21] and moderate predictive ability in identifying patients with spinal pain at risk of persisting pain and disability [22]. The questionnaire contains 25 items, and items 5–25 are scored [23]. Lower cut-off limits for ÖMPSQ were 89 for medium risk and 112 for high risk (corresponds to 42 and 53% of total score). Based on a recent study, we chose to omit the work questions in the ÖMPSQ total score for non-working patients [24]. Therefore the five work-related items no 6, 8, 16, 17, and 20) were excluded for participants out of work, and new scoring range and cut-off values were calculated based on the percentage of total score omitting five variables (i.e. 42 and 53% of 160). This gave cutoffs of 67 and 85 for medium and high risk classification among non-workers, respectively.

### Analysis

**Fig. 1:**
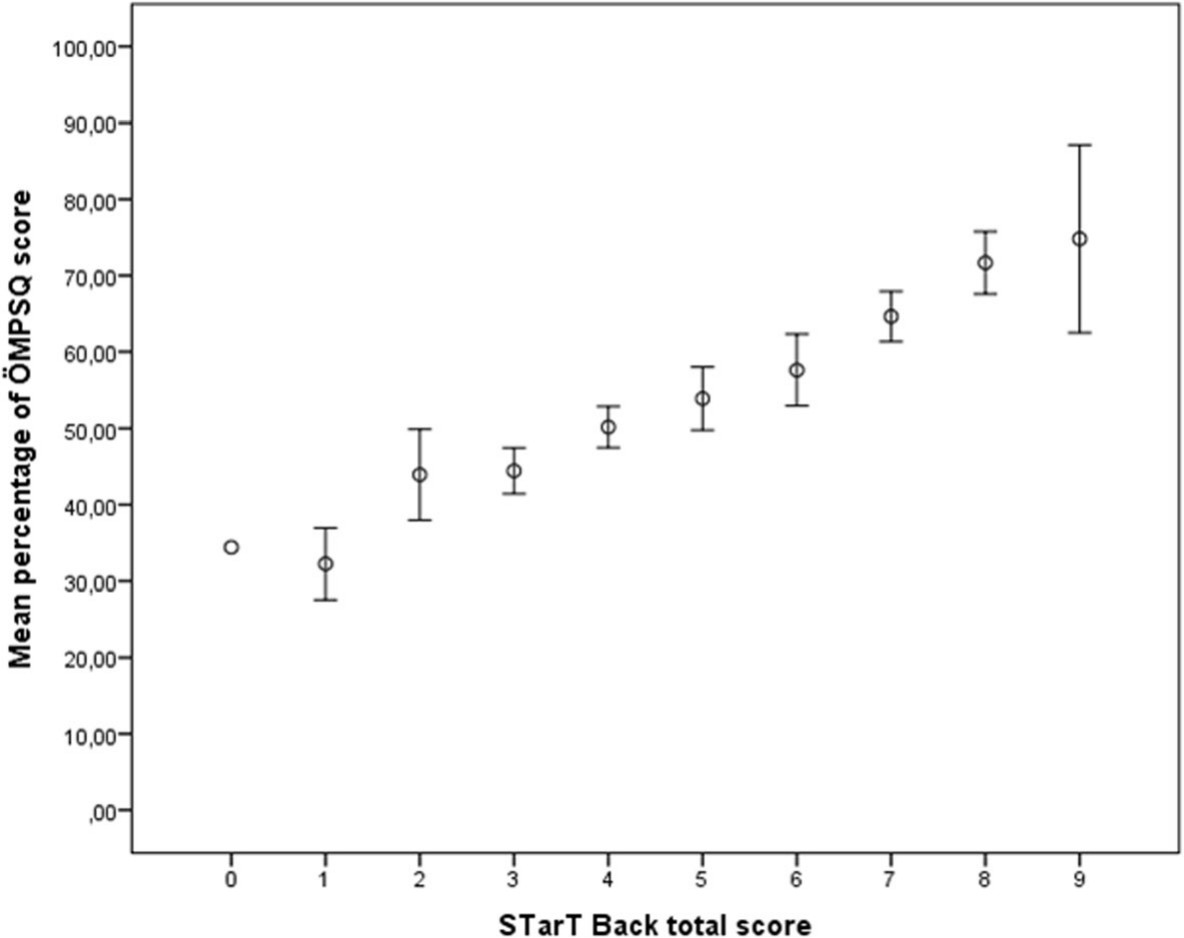
Mean percent of Örebro Musculoskeletal Pain Score Questionnaire (ÖMPSQ) according to Start Back total score. Vertical bars represent 95% confidence interval. (Pearson’s *r* = 0.76)

To assess criterion validity of STarT Back, the agreement in risk classification (low, medium, and high) based on STarT Back and ÖMPSQ was assessed by Cohen’s Kappa coefficient. The calculations were done for the overall study population, but since some of the items of the ÔMPSQ are work related, we also estimated the agreement in risk classification for patients who were classified as workers. We also calculated *the mean* ÖMPSQ score with 95% confidence interval (CI) according to *STarT Back* total score (Fig. 1)*.* Since the two scores are measured on different scales, we converted both scores to percentage scores before we used Pearson’s *r* to estimate the correlation between STarT Back and ÖMPSQ. The acceptability limits were defined as: poor ≤30; adequate 0.31–0.59, and excellent ≥0.60 [25]. The percentage scores were also used in a Bland-Altman plot assessing the agreement between STarT Back and ÖMPSQ screening tools.

Risk classification of the patients, as indicated by both instruments was described by a classification table. Finally, we used linear regression to estimate mean differences with 95% CI in pain and work related variables between the three risk groups defined by the STarT Back tool. Non-normally distributed variables were analyzed using non-parametric Mann-Whitney U test and results presented as differences in median values between risk groups.

## Results

A total of 300 patients received the study questionnaire, and 199 (66%) returned it. After excluding 17 persons due to incomplete answers or age below 18 years, 182 patients (61%) were available for statistical analysis. Among those, 73% were employed, mean age was 48 years (SD = 15), and 51% were men (Table 1).

**Table 1:** Characteristics of study population

Variables			STarT Back risk groups					
	Total		Low		Medium		High	
	*N* = 182		*n* = 62		*n* = 77		*n* = 43	
Age^a^	48	(15)	47	(15)	50	(15)	47	(14)
Gender, men^b^	92	(51)	29	(47)	40	(52)	23	(54)
Other diseases^b^	127	(70)	39	(63)	57	(74)	31	(72)
Musculoskeletal comorbidity^b^	68	(37)	17	(27)	33	(43)	18	(42)
Married^b^	97	(53)	30	(48)	43	(56)	24	(56)
Born in Norway^b^	169	(93)	60	(97)	73	(95)	36	(84)
University / college education^b^	77	(42)	31	(50)	32	(42)	14	(33)
Employed^b^	133	(73)	51	(82)	55	(71)	27	(63)
Sicklisted^b^	58	(32)	16	(26)	26	(34)	16	(37)

^a^Mean, SD

^b^N, percent

**Fig. 2:**
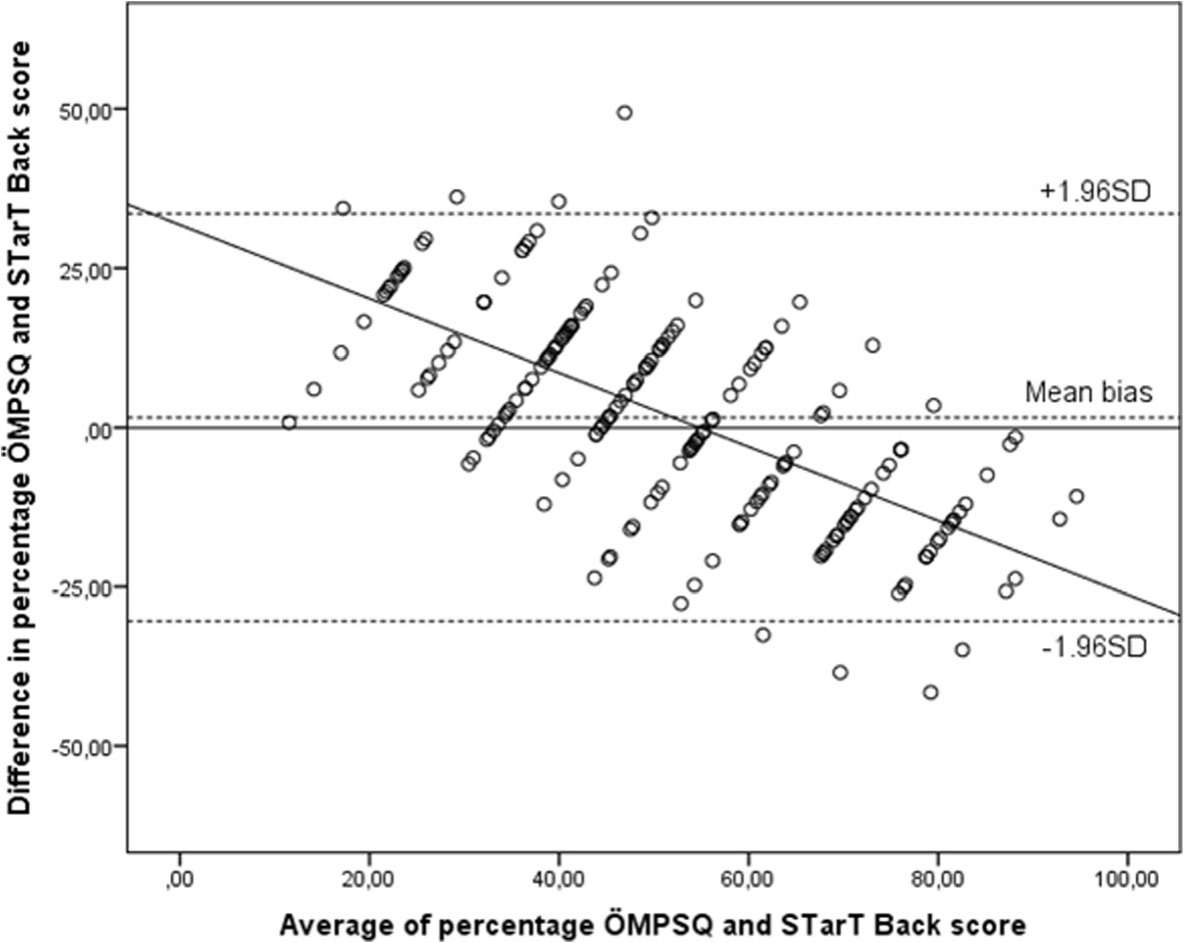
Agreement between STarT Back screening tool and Örebro Musculoskeletal Pain Screening Questionnaire (ÖMPSQ). Dotted lines in the Bland-Altman plot represent mean bias with 95% limits of agreement. The solid diagonal line represent the difference between the screening tools regressed on the average of the two tools (slope = − 0.58). X-axis displays average percentage score between both instruments. Y-axis display differences in percentage score

The Kappa value for agreement between risk group classification was 0.35 (Table 2). Restricting the sample to only workers gave the same level of agreement (Kappa 0.36). Figure 1 shows that mean percentage of total ÖMPSQ-score increased monotonically with increasing STarT Back total score, and the two scores showed a high correlation (*r* = 0.76, p˂0.001). The Bland Altman plot (Fig. 2) display the difference between the two instruments according to the average percentage scores for both instruments, and suggests that the agreement between the instruments is highest for middle range scores. Mean bias is 1.6, but for patients scoring in the lower range on both scores there is tendency that STarT Back generates lower score than ÖMPSQ. On the other hand, in patients with higher average scores, STarT Back seems to generate higher scores than ÖMPSQ.

**Table 2:** Classification table showing agreement between risk group stratification as defined by STarT Back and Örebro screening tools

	Örebro Musculoskeletal Pain Questionnaire risk group				
Start Back Screening tool risk group	Low	Medium	High	Total	Percentage
Low	35	22	5	62	34.1
Medium	9	27	41	77	42.3
High	1	3	39	43	23.6
Total	45	52	85	182	
Percentage	24.7	28.6	46.7		

Kappa coefficient = 0.35

Start Back Screening tool allocated 34.1% of the participants in the low risk group, 42.3% in the medium risk group, and 23.6% in the high risk group. Corresponding values for ÖMPSQ was 24.7% of the participants in the low risk group, 28.6% in the medium risk group, and 46.7% in the high risk group (Table 2). In supplementary analyses restricted to workers only, 38.3% were allocated in the low risk group, 41.4% in the medium risk group, and 20.3% in the high risk group as defined by STarT Back.

There was a dose-dependent relation between scores on pain and work items on ÖMPSQ and STarT Back risk group (Table 3), except for the item on job satisfaction. Pain variables differed most between low and medium risk group, while work variables separated most clearly between the medium and high risk group as defined by STarT Back.

**Table 3:** Mean differences from linear regression analyses^a^ of selected pain and work related variables between Start Back risk groups (work related variables analyzed only on people in work, and higher scores represent worse problems)

	STarT Back risk group		
	Low risk	Medium risk	High risk
Pain			
Intensity last week, 1–10 points			
Mean (SD)	3.8 (2.0)	5.1 (2.5)	5.7 (2.7)
Difference (95% CI)	0.0 (reference)	1.2 (0.3–2.1)	1.9 (0.8–2.9)
Intensity past 3 months, 1–10 points			
Mean (SD)	6.0 (1.5)	6.9 (1.5)	7.9 (1.5)
Difference (95% CI)	0.0 (reference)	1.0 (0.4–1.5)	1.9 (1.2–2.6)
Duration, weeks			
Median (IQR)	46 (20–52)	52 (33–52)	52 (47–52)
Difference (*p*-value)	0 (reference)	8 (0.007)	8 (0.004)
Work			
Missed days of work, passed 18 months			
Median (IQR)	11 (0–137)	30 (5–137)	137 (60–365)
Difference (*p*-value)	0 (reference)	19 (0.14)	126 (0.001)
Heavy / monotoneous work, 1–10 points			
Median (IQR)	5 (3–7)	7 (5–8)	8 (6–9)
Difference (p-value)	0 (reference)	2 (0.02)	3 (0.004)
Workable in six months, 1–10 points			
Median (IQR)	1 (0–5)	2 (0–5)	6 (2–9)
Difference (p-value)	0 (reference)	1 (0.13)	5 (< 0.001)
Job satisfaction, 1–10 points			
Median (IQR)	2 (1–4)	2 (1–5)	3 (1–5)
Difference (p-value)	0 (reference)	0 (0.23)	1 (0.17)
Should not work (fear), 1–10 points			
Median (IQR)	4 (0–6)	5 (3–9)	10 (6–10)
Difference (p-value)	0 (reference)	1 (0.01)	6 (< 0.001)

^a^Non-normally distributed variables were analyzed using non-parametric Mann-Whitney U test and results presented as differences in median values between risk groups

## Discussion

This study indicated high correlation between instrument scores and low agreement between risk classification between StarT Back tool and ÖMPSQ for patients referred to secondary care because of low back pain. Risk group classification according to STarT Back allocated 23.6% in the high risk group. According to ÖMPSQ, 46.7% were allocated in the high risk group. Patients classified with high risk according to Start Back showed a higher score on pain and work related characteristics as measured by ÖMPSQ.

The Start Back total score highly correlated with ÖMPSQ total score, indicating good criterion validity for STarT Back in a Norwegian sample of low back pain patients referred to a multidisciplinary outpatient clinic in secondary care. This result is comparable with previous studies performed in other countries and settings. Bruyere and coworkers [10] addressed the correlation between the ÖMPSQ and STarT Back and found a Spearman correlation coefficient of 0.74. The latter study included patients in settings different from the present study; a rehabilitation center, a back school, a private physiotherapy unit as well as persons with low back pain at a fitness center [10]. ÖMPSQ has also been compared to STarT Back screening tool in England, including two hundred and forty-four consecutive non-specific low back pain consulters at general practitioners [19]. They found a correlation between STarT Back tool and ÖMPSQ of 0.80 and classification agreement Kappa 0.57. Significant differences between STarT Back and ÖMPSQ-registered threshold were observed in that STarT Back allocated fewer patients to high risk classification.

Despite very good correlation between the two scales, the findings from the risk classification in the present study showed that 22 out of the 52 patients classified as medium risk by ÖMPSQ were classified as low risk on Start Back. Additionally, 41 out of the 85 patients with high risk according to ÖMPSQ were classified with moderate risk according to Start Back, in line with the study from England [19]. One may ask whether these discrepancies were related to the fact that ÖMPSQ has five work-related questions and have been suggested to be a good predictor of future absenteeism [18], while Start Back does not include any work questions. Studies assessing the validity of the short-form ÖMPSQ that includes ten items, of which two covers work that is optional connected to the home or to paid work may support this hypothesis as it showed less discrepancies in classification when compared to STarT Back [17, 26]. Because STarT Back does not cover work, we hypothesized that STarT Back could underestimate risk for participants with work-related obstacles for recovery. Our results showed that patients classified with high risk according to Start Back showed a significantly higher score on work related characteristics as measured by ÖMPSQ, with one exception for the item on job satisfaction. These findings suggest that specifically screening for work factors is important in this group of patients. This is also indicated by the difference in ÖMPSQ-scores between workers and non-workers, in line with results from a recent factors analysis [27]. Further research is needed to confirm this relation, and to address the need for more knowledge regarding referral practice and the right candidates for multidisciplinary rehabilitation to restore employability [28].

Another finding was that the agreement for score was best for middle range scores, and that the compliance between tools were lower in both ends of the scoring scales. For the higher mean percentage scores, the tendency was that STarT Back tool allocated higher score. In spite of that, Örebro allocated a higher percentage of participants to the high risk group, again indicating a lack of correspondence between the cut-off limits for the instruments.

The results indicated that between 24.7 (ÖMPSQ) and 34.1 (STarT Back) percent of the respondents had low risk for longstanding disability due to low back pain. Clinical guidelines recommend secondary care referral when management needs are too complex for primary care management [4]. Given that, it is somewhat surprising that as much as 25–34% of the patients were classified low risk. Multidisciplinary management in secondary care is the recommended treatment choice for patients with significant obstacles to recovery and / or when previous treatment have not been effective [5]. In general, secondary care referral may be due to complex psychosocially oriented treatment needs or to suspicion of organic disease from the primary care contact. The outpatient clinic in the present study offers multidisciplinary treatment targeting psychosocial needs as well as examination of potential pathoanatomic triggers. Therefore, screening tools designed for early addressing of psychosocial obstacles for recovery in primary care may not be sufficient or relevant to consider if a patient should be managed in secondary care.

To our knowledge, no studies have compared scores and classification by these two screening instruments in patients referred to secondary care. On the other hand, the predictive value of both instruments in secondary care has been evaluated. An Australian study concluded that the instruments add no further value over and above clinical judgement [29], and a Danish study concluded that the predictive ability of STarT Back is less good in secondary care compared to primary care [30]. Again, it is plausible to suggest that the most relevant screening items for primary and secondary care patients differ.

### Strengths and limitations

This is a cross-sectional study, and it cannot evaluate the predictive value of the screening instruments. The response rate was 66% and we cannot rule out that the study participants were different from the non-responders. Consequently, the results may not automatically be generalisable to the population of patients referred to the multidisciplinary clinic. Missing responses on some items may have introduced bias. To compensate for missing on the work items, supplementary analyses were performed to compare whole-sample results to results from analyses restricted to patients in work.

The results from the present study do not suggest that the risk classification by STarT Back is comparable to risk classification by ÖMPSQ for patients referred to secondary care for low back pain, though the instruments’ scores correlated well. This study also suggests that around one third of the patients referred to secondary care will be classified low risk according to these primary care screening tools. The results do not support the applicability of StarT Back screening tool as decision support in Norwegian secondary health care. This may be due to differences in timing of testing, clinical setting, and study sample compared to the original target group for screening. The results also indicated that the working items in ÖMPSQ may be central when addressing psychosocial load in working patients.

## Conclusion

STarT Back scores correlated well to scores on ÖMPSQ, while classification agreement between the instruments was low in patients referred to multidisiplinary secondary care for low back pain. Patients classified as high risk by STarT Back reported more challenges connected to work on ÖMPSQ sub-items.

## Abbreviations

CI: Confidence interval
ÖMPSQ: Örebro Musculoskeletal Pain Screening Questionnaire
STarT Back: STarT Back screening tool
